# Publishing peer review materials

**DOI:** 10.12688/f1000research.16460.1

**Published:** 2018-10-17

**Authors:** Jeffrey Beck, Kathryn Funk, Melissa Harrison, Jo McEntyre, Josie Breen, Andy Collings, Paul Donohoe, Michael Evans, Louisa Flintoft, Audrey Hamelers, Phil Hurst, Thomas Lemberger, Jennifer Lin, Niamh O'Connor, Michael Parkin, Sam Parker, Peter Rodgers, Magdalena Skipper, Michael Stoner

**Affiliations:** 1 National Center for Biotechnology Information, National Library of Medicine, National Institutes of Health, Bethesda, MD, 20894, USA; 2 eLife Sciences Publications, Ltd, Cambridge, UK; 3 European Bioinformatics Institute (EMBL-EBI), Wellcome Trust Genome Campus, Hinxton, Cambridge, UK; 4 BMJ, London, UK; 5 SpringerNature, London, UK; 6 F1000, London, UK; 7 The Royal Society, London, UK; 8 EMBO, Heidelberg, Germany; 9 Crossref, Oxford, UK; 10 PLOS, Cambridge, UK; 11 PeerJ, London, UK

**Keywords:** peer review, scholarly publishing, JATS, JATS4R, Crossref

## Abstract

Publishing peer review materials alongside research articles promises to make the peer review process more transparent as well as making it easier to recognise these contributions and give credit to peer reviewers. Traditionally, the peer review reports, editors letters and author responses are only shared between the small number of people in those roles prior to publication, but there is a growing interest in making some or all of these materials available. A small number of journals have been publishing peer review materials for some time, others have begun this practice more recently, and significantly more are now considering how they might begin. This article outlines the outcomes from a recent workshop among journals with experience in publishing peer review materials, in which the specific operation of these workflows, and the challenges, were discussed. Here, we provide a draft as to how to represent these materials in the JATS and Crossref data models to facilitate the coordination and discoverability of peer review materials, and seek feedback on these initial recommendations.

## Introduction

Peer review is the practice of subjecting a scholarly article, such as a research paper submitted to a journal, to scrutiny or review by others ('peers') who are experts in the same field. Generally, if the author of the article addresses the concerns raised during peer review to the satisfaction of an editor, the article is accepted for publication. The peer review process produces a trail of documents, which can include: different versions of the article; the reviewer reports (with or without the name of the reviewer); responses by the author to the reports; and various letters (including cover letters from the author and decision letters from the editor). It is also possible for an article to go through two or more rounds of peer review, which increases the number of documents generated.

Traditionally the documents generated during the peer review process were only ever seen by the author, the editor and the reviewers, but a small number of publishers now publish some peer review materials alongside articles. Moreover, support for this practice has been slowly gaining momentum, driven by a wish to increase transparency and provide credit for peer reviewers ( Polka *et al*., 2018). There were 10 journals identified in the PMC corpus that archive some peer review materials. These journals take a variety of different approaches, which results in differing levels of discoverability for these materials. Additionally, some journals were identified as publishing peer review materials but not consistently archiving them in a repository.

Here we report the findings of a workshop, held at the BMA in London, UK on July 6, 2018, at which representatives from publishers, PubMed Central (PMC)/Europe PMC, and Crossref discussed the practical challenges involved in publishing peer review materials. We focus on what has to happen after a publisher decides to start publishing peer review materials, and discuss how to do this in a way that is sustainable, improves discoverability, and supports machine readability and archiving. We do not discuss the relative merits of the different approaches to peer review that have emerged over the past decade, notably the many different flavours of 'open peer review' ( Ross-Hellauer, 2017), but we feel that many of our suggestions and recommendations are relevant to most if not all of these approaches.

## What are peer review materials?

As mentioned above, peer review materials can include: the reviewer reports (with or without the reviewer name); responses by the author to the reports; and various letters (including cover letters from the author and decision letters from the editor). Some articles go through two or more rounds of peer review, which increases the number of documents generated. While each document is usually accompanied by a date, other metadata concerning the correspondence can be highly variable. For example, peer review reports and editors decision letters may or may not include names of reviewers or editors, or their ORCID IDs; the individual materials may or may not have DOIs. In subscription journals it is also possible for peer review materials to appear in front of or behind the paywall.

Publishers are approaching the publication of peer review materials in a number of ways. The aims of this group were not to prescribe what should be done from an editorial point of view, but to enable what is published to be found by readers and machines alike. Prior to the workshop, data were collected from each publisher attending and we found the following materials are published:

○ Peer review reports, anonymized or with report author names
○ Author responses/Rebuttals
○ Editor decision letters

Some journals also provide appeal and resubmission information (including previous versions of the article, dates, and actors involved).

In some cases publishers make peer review materials available as a single PDF with versioned reports linked to specific revisions of the article. Other publishers create separate artifacts, each with unique DOIs, and still others edit and amalgamate various reports into one narrative. The variety can be found in the meeting notes and a table filled out before the meeting (See Supplementary File 1 and Supplementary File 2).

After collecting these data, representatives from each of the identified publishers were contacted to attend a workshop in London on July 6, 2018; all but one publisher was able to attend. Further publisher representatives were invited following communication with ASAPbio – these publishers are embarking on this practice. Crossref, Europe PMC and PMC were also represented at this meeting, as downstream recipients of this content or the metadata related to it. Journal representatives expanded on the data previously collected and shared details on how they collect and publish peer review materials, how these artifacts are represented in the ANSI/NISO Z39.96-2015 Journal Article Tag Suite (JATS) document standard (these principles can be applicable to other DTDs), and whether they send relevant metadata to Crossref. Crossref shared details of their schema extension to represent this form of content ( https://www.crossref.org/services/content-registration/peer-reviews/). As downstream recipients of peer review materials, PMC and Europe PMC presented the perspective of archives that collaborate with journals to ensure that content is being captured in a sustainable and consistent format that fosters long-term preservation and access to the scholarly record. Understanding the goals of, workflows, and limitations on each stakeholder allowed the group to refine the scope of the discussion and its outcomes.

## Peer review materials need to “stand alone”

In order to advance the transparency and recognition of peer review materials, we agreed these peer review materials need to stand alone from the main article for the purposes of, for example, credit and citation. Ideally, each content item should have its own DOI (as per the recently enhanced Crossref schema). We identified three levels of achieving this, with level 1 being the basic and least preferred option, but probably currently the most achievable and pragmatic:

1. Peer review materials are attached to the article as a single or numerous PDFs. Whether these materials are pulled together into one document or attached as separate documents, there should be some defined mechanism in the JATS XML tagging that would support the capture of any available metadata and identify these files in a machine-readable and interoperable way for publishers to tag this content appropriately.
2. Peer review materials are appended to the article within the full text (so all is machine readable) as a sub-article component of the XML.
3. Peer review materials are full-text XML “articles” or “commentaries” in their own right that link bidirectionally to the main article.

## Required metadata versus rich metadata

Whether the material is provided as a PDF(s) attachment to the main article, or as a full-text XML sub-article or separate article, important metadata can be attached to the item in a machine-readable way, and DOIs can be applied. What types of peer review information are available is dependent on the publisher’s peer review policy, for instance whether reviewers and editors are named, whether the peer review material carries the same license as the main article or takes another form, and what items constitute the peer review materials. Additional metadata fields, such as dates of review and date of review publication and the inclusion of ORCID IDs for reviewers and editors will also be subject to publisher policies and workflows. However, all of this material can be added to the item in a machine-readable way. Even if the actual content is not published in full-text XML format, the metadata can (and should) be.

The topic of licensing of the peer review materials was briefly discussed at the workshop but ultimately left out of the remit of this group because the JATS tagging schema would allow for different licensing information to be added for these items or to retain that of the main article, as a publisher chooses.

## Challenges in the process

While a few of the publishers had processes in place to prepare the peer review content automatically or within a few minutes, others spend 20–40 minutes per article. In such cases, the tasks that are attributed to this time include the following:

- Removing boilerplate text from review reports
- “Stitching together” the material from disparate locations in the submission system
- Editorial checks:
  - Reviewing the content for sensitive information, e.g., unpublished data additions and confidentiality leaks, as well ensuring the tone of the report is appropriate
  - Removing author responses that contain data the author wants to publish in a subsequent paper
  - Arbitration processes for conflicting reviews

Where time is spent—whether in the editorial or production process—depends on the publisher workflow. Regardless of workflow, the overlap in tasks identified provides evidence of the potential value of updating the infrastructure of submission systems to account for and streamline these efforts. Coordination between publishers and submission systems could minimize the time spent “stitching together” peer review materials into a publishable format.

In addition to time and workflow hurdles, another major challenge noted by those publishers without their own hosting platforms, was the actual publication process and online hosting of peer review materials. Many publishers identified that some online hosts were not able to manage this new content type. As a result, peer review materials are being captured in supplementary material sections because alternative options are not available. In such cases, it becomes more difficult to capture any relevant associated metadata in a meaningful way for the peer review materials or to make this valuable content easily discoverable.

These challenges are common for publishers in that most of the established submission systems and hosting platforms were designed and built many years ago and may be slow to accommodate new requirements. Coordinated communication with these platforms regarding the workflows around publishing peer review materials may result in more satisfactory and generic approaches to accommodating publication of peer review materials.

There are internal challenges of cost control issues that also need to be accounted for, and the publication of a single PDF is often more achievable financially based on current systems than producing full-text XML. However, the attachment of machine-readable metadata to that PDF should be within reach, especially if the submission systems and hosting platforms can build these requirements into their products.

## Importance of version management

An additional challenge may be introduced in managing peer review materials in cases where such materials are collected for more than one published version of a paper. The Recommendations of the NISO/ALPSP Journal Article Versions (JAV) Technical Working Group ( 2008 April), included the following types of article instances:

- authors-original
- submitted-manuscript-under-review
- accepted-manuscript
- proof
- version-of-record
- corrected-version-of-record
- enhanced-version-of-record

To this list, the JATS4R working group on “Article publication and history dates” added pre-print. The JATS4R draft recommendation advises that if the publisher publishes a revision of any of these stages, the subsequent revisions should be labelled with suffixes, as follows: “-r1”, “-r2”, etc. ( https://jats4r.org/article-publication-and-history-dates).

If the peer review materials reference content in a specific version of an article, that link between peer review materials and correct version should be captured in the metadata for clarity. Managing the associations between peer review materials and article version is essential for journals that make multiple versions of a paper publicly available, to ensure the archival record is accurate and that the process transparent. For example, if a journal publishes three versions of an article, any related peer review materials should be associated with the appropriate version. It should not be left to a reader to determine if a peer review report or decision letter relates to the first version, the second version, or the third version.

## JATS XML proposal (designed to aid depositing to Crossref)

Irrespective of the editorial and publisher decisions regarding workflow, we propose the following options regarding JATS XML tagging, designed to also aid metadata registration with Crossref (note we are using the same terms as Crossref where controlled vocabulary is required).

### Overarching document type

Review documents may be supplied as:

1. sub-articles <sub-article> to the article being reviewed (sub-articles may be full-text XML or XML metadata plus a link to the PDF)
2. independent articles <article> (with the appropriate <related-article> links – Peer Reviews MUST link to the version of the article they are reviewing and Author Replies; Decision Letters MUST link to the version of the article they are passing judgment on; and Author Replies MUST link to each Review/Decision Letter it is addressing)

### Identifying the type of content

<sub-article> or <article> MUST have an article-type attribute with one of the values listed in Table 1.

**Table 1.** Article-type attributes.

Attribute (as per Crossref schema)	Corresponding term in this document
referee-report	Peer review
editor-report	Decision Letter
author-comment	Author Response/Rebuttal
aggregated-review-documents	Collected Review Documents

**Note**: aggregated-review-documents is not currently in the Crossref schema; that schema uses the term aggregate. Crossref has two further attributes to describe the type of content: **community-comment** and **manuscript.** The XML sub-group discussed these terms and decided to exclude them as community-comment presumably refers to post-publication comments via systems like Hypothesis and so: a) are not guaranteed to be “peer” comments and are excluded from the criteria of this paper and b) it is unlikely that publishers in the near term would pull that content back into the source JATS XML, post publication. Crossref schema also allows for a stage, pre-publication or post-publication. This is therefore also felt outside of this remit.

The term manuscript does not map to anything we’ve discussed.

### Identifying the recommendation

This is an optional item. Currently there is no corresponding tag in JATS and so would require a request to the JATS Standing Committee.

There would be a fixed value list, mapped to the Crossref schema:

major-revision

minor-revision

reject

reject-with-resubmit

accept

accept-with-reservation

NOTE: There should be no “recommendation” for author-comment type content.

### Identifying the authors (including ORCIDs)

It is an optional element and should be contained within <contrib>, which should contain a <name> or <anonymous/>.

If <contrib> is used, it MUST contain @contrib-type that maps to following controlled vocabulary:

○ For Peer Reviews, use @contrib-type=“reviewer”
○ For Decision Letters, use @contrib-type=“editor”
○ For Author Reply, use @contrib-type=“author”

We intend that the @contrib-type attribute value reflects the contributor’s relationship to the peer review process and not the relationship with the document.

The <role> tag is optional and can be used for display terms of what publishers may use for their journal (for example variations on the term editor could be Academic Editor, Reviewing Editor, Senior Editor, E-i-C etc.).

Names, affiliations and contributor IDs (such as ORCID), where provided, follow standard JATS tagging (see JATS4R recommendation: https://jats4r.org/authors-and-affiliations).

### Identifying any competing interests

Follow tagging recommended by JATS4R: https://jats4r.org/conflict-of-interest-statements.

### Identifying DOIs

DOIs for peer review materials are optional but strongly encouraged. Use <article-id pub-id-type=“doi”>

### Licensing and copyright

Each review document (standalone article or sub-article) SHOULD have license information with a machine-readable license. Review documents supplied as <sub-article> may have their own <license> element or inherit their license information from the parent document as described in the JATS4R Permissions Recommendations ( https://jats4r.org/permissions).

### Date

Each review document (standalone article or sub-article) MUST have a pub-date and may have other publication information captured as <event>. Review documents supplied as <sub-article> may have their own <pub-date> element or inherit their <pub-date> from the parent document.

### Not allowed

There are some elements that MUST NOT appear in review documents:

a. <funding-group>
b. <app>, <app-group>, <ack>, <glossary>, <back>/<sec>
c. <supplementary-material>, <inline-supplementary-material>
d. <bio>
e. <article-version> Once published, review documents will not be “versioned”. If the reviewer(s) write a review on an updated version of the manuscript, the peer review is a new published object.

## Crossref metadata

As of November 2017, Crossref supports the scholarly discussions entailed in the publication process as well as those after publication (e.g. “post-publication reviews”). In the same fashion as all content registered with Crossref, peer review metadata is available via the open Crossref APIs and Crossref Metadata Search. For full details and example deposit XML, see the Crossref peer review deposit guide: https://support.crossref.org/hc/en-us/articles/115005255706-Peer-Reviews.

## Display of peer review materials

We also propose that publisher web platforms and archives display peer review materials (or links to peer review materials) in a clearly labeled peer review section. This practice will help ensure that not only are the journal processes transparent but that the content itself is easy to find and navigate to, regardless of how a journal chooses to make them available.

## Future/next steps

This proposal is intended to lay the groundwork for the publication and archiving of peer review materials across publishers and publication models, providing flexible options to meet different journal needs and workflows. Moving forward, there is a need for continued collaboration and discussion as peer review models and workflows evolve. As the goals of these peer review efforts are more clearly defined across the publishing and academic communities, certain models may lend themselves more readily to supporting those desired outcomes. Continued efforts to identify the most critical needs of each user group should be explored through ongoing efforts such as ASAPbio and FORCE11. In turn, these needs can inform the technical solutions and recommendations going forward.

Further technical discussions should not be placed on hold in the interim, though. As publishing peer review materials practices grow, there is a pressing need for industry-wide solutions now. We would like to see the XML recommendations from this group be converted to a JATS4R recommendation on the publishing of peer review materials. Similarly, it would be of value to the community for Crossref and JATS to coordinate efforts and ensure some level of metadata alignment for peer review materials that would reduce costs to publishers and minimize barriers to implementation.

This type of coordination between publishers, archives, and other organizations that support the scholarly communication enterprise is critical to ensuring that the needs of the whole community are being met. Past experience has taught us that making content available is just the first step toward increasing transparency. Doing so in a flexible, consistent, and meaningful way is imperative in making certain that the available material is also discoverable and that long-term preservation of the content can be supported. Implementing the next steps through community-driven recommendations in a sustainable way will be important in increasing transparency and rigor of the scientific record.

If you are publishing peer review materials, or are not yet and are considering doing so, please comment.

## Data availability

No data are associated with this article.

## Supplementary material

**Supplementary File 1. Workshop agenda and notes from the meeting.**

**Supplementary File 2. Current processes of the different journals represented at the meeting.**
