# Supplementary material for: Publishing peer review materials
Source: F1000Res. 2018 Oct 17;7:1655. [Version 1] doi: 10.12688/f1000research.16460.1 (PMC6206614; doi:10.12688/f1000research.16460.1)
Supplement: Supplementary file 1 [file f1000research-7-17992-s0000.tgz › 023285ab-1492-4902-acad-db913e9621a4_Supplementary_file_1_workshop_agenda_and_meeting_notes.docx]

**Publishing Peer review materials workshop**

**Background**: PMC identified 9 participating journals which are providing peer review reports/material in their published output. Each journal is doing so differently and the material is not always machine readable. Linked to this, Crossref have a new model whereby publishers can register DOIs and metadata regarding the peer review materials they publish.

Eight of these 9 journals (excludes Wellcome as it follows the F1000 model; GigaScience cannot make it), Crossref and representatives of NCBI and EBI have agreed to participate in this workshop to discuss this.

**Aims of meeting:** Each publisher to present their model and discuss their reasons and motivations for this model. To build consensus on consolidating to 1 model, or at least a few tiers of a single model, and begin the investigation to suggested tagging within the JATS DTD.

**Agenda**:

9.30 Welcome and round-table introductions

9.40 Agreeing the goals of workshop

10-12 Go round the table - each publisher to speak to their line/experiences

1. BMC
2. BMJ
3. eLife
4. F1000
5. Nature Communications
6. PeerJ
7. PLOS
8. Royal Society
9. EMBO?
10. Crossref
11. EPMC/PMC landscape overview

12-1 Lunch

1-3 Group discussion – consensus to a common model/framework

3-4.30 Group discussion – potential JATS XML data model

Next steps

**Potential outcomes**:

- Write up of the discussions in the form of a paper submitted to appropriate venue
- JATS4R recommendation
- Moving the consensus to another organisation such as AsapBio or Force11 to promote the decision to the wider publishing community

Resources -

- [Current publisher implementation spreadsheet](https://docs.google.com/spreadsheets/u/1/d/1_-SGubM_4mXBX3Fcx1xJ4C6GEGijTnzYkg8M2WJLa6k/htmlview#gid=0)
- [Crossref metadata for peer review reports](https://docs.google.com/spreadsheets/d/1Ib_KEkLfNoPcZozjJlMVvQxHFv-dJB3dU1MIITvUAuA/edit#gid=0)

**Meeting**

Common vision best practice

Versions

Searchability

Openness

Edited/Unadulterated

Cascade from other journals internal and externals

Inappropriate comments in reviews

Licensing?

Clear up from with your reviewers - reviews moving between publishers and journals - copyright etc...CC-BY

Author decides whether referee reports open or not and so referees not given choice if author says no

Concrete outcome - source of reference for people thinking about these things

**Round table**

*eLife*

Publish 100 research papers per month

Decision letter and response each published - full text as separate entities

6 years ago, could opt out, but 1% did opt out

Good feedback

Sometimes publish seperate reviews, but not tagged separately

Sometimes authors slide in unpublished data, confidentiality revealed

Summary - major comments, omit minor comments (ei typos)

Don’t require reviewers to self-identify but encourage 30% do

Repetition in author response - extracts from decision letter

Full text XML

Sub DOIs

~30 min production time per decision letter

*EMBO*

Since 2009

Less than 1% opt out

Publish reports - review process file, single document, PDF

Title of paper, link to paper, date of publication, timeline of review process incl appeals/resubmissions/name of handling editor

Sequence of docs in chronological order - letters and review reports, reply by author, cyclic

Untouched but letters are edited to remove boilerplate text

If reply contains data that author wants to publish in in subsequent paper, will be removed

Pre submission consultation - editor paraphrases

Reviewers anonymous - welcome identification, but up to them. No structure to capture this

Production - copy and paste into a database

Takes 30 min +

Editor checks accuracy of what’s assembled.

Point by point responses from authors - Word file with lots of “decoration”

Arbitrator - complicated - two conflicting reviewers

*Royal Soc*

Started 2014 - royal soc open science

Author option to publish

Referees could opt out knowing it was open

Published peer review reports, decision letters and author responses

Opt in via ScholarOne - metadata exported into production system. Checks carried out and published as a PDF. Checks take 40 min

Don’t add DOIs - host platform limitation

Open Biology journal- mirrors embo model

Looking to extend open peer review to more journals

Opt in is fiddly - mandate it, it’s easier. For publishing of reports

Transfers from other journals (internal other journals)

*BMC*

45 journals publishing reports since 2000

Mandatory

Mandatory reviewers sign reports

But it decreases reviewer rates and reviewer pool diversity - so re-thinking required openness

Reports pulled from the production system and the author responses

Put into xml - each report and each response is on separate page

Previous versions of manuscript, in theory, but has to be by request and can be hard to get them

Would like DOIs for the reports, but not technically possible right now. Waiting for JFlux

Main article link to peer review reports, page with table on it, that links to page for each of these things

*Nature Communications*

Mandatory peer review

Reviewers can sign if they wish

Single PDF "Peer Review File" report containing review report (anonymous), author response, editor name and comments. inserted into Supplementary Information section.

Shoe string - document is very primitive

Way of getting around internal restraint

Lot of appetitive to publish ref reports on non-open access journals

Physical Sciences are not ready for mandated open peer review

*F1000*

Post pub open peer review

Referee names open and 3 statements: Approved, Not Approved, Approved with Reservations

Ref reports have DOIs

Mint article DOIs with crossref and related

Reviews are datacite DOI but article are Crossref DOIs

No editorial decision letter

Authors can respond and goes into the page but they don’t get a DOI

Suggesting referee names is author led - unusual and can be hard to get them

Editorially checking the names for CoIs

*BMJ Journals*

BMJ Open and BMJ Open Science

PDF - ScholarOne, lots of cut and paste

HighWire hosting restrictions

One PDF - reviewer comments, author response, V1, V2

BMJ

More PDF documents - each version split out

Open peer review - only research and analysis articles

Reviewers sign their reviews.

Patient reviewers also sign, but offered anon if specifically requested..

All PDFs cut and pasted from scholarone and manually upload to website

30-45 min per paper (publishing 4 research articles per week)

*PLOS*

Don’t publish any peer reviews currently but want to start doing it

Discussions and intentions:

Publish reports and decision letters - publish as much as possible, considering removing boilerplate “stuff” from the letters

Exact way the process will work is under decision

Opt in for reviewers to sign

Authors opt in if they want to make peer review materials available

Hope opt in will be high enough so can flip to mandatory

Finalising whether all or some journals will publish review materials initially

Journals with academic editors (rather than in-house) have different challenges for setup as need support of Editors-in-Chief

Would like to do for PLOS One

*PeerJ*

Full time line

Reviewers fill in 4 areas and captured

Very clean and captured easily but also allows reviews submitted as a PDF

Whole process happens as it goes, so not doing it all at the end

Public review and reviewer wants to be anonymous have to allow for that

Rebuttals etc are downloadable

Register reviews and preprints with Crossref

Don’t register the editorial decisions and letters

Write site themselves and do it all inhouse - all automated

*PMC/Europe PMC*

Aggregator and archive: want peer reviews to be archived in a sustainable, sensible way

Tracking who is sending them Peer review is really challenging

So many ways and so many formats

Mostly PDFs reviews pre-publication

Different models, different implementations , lots of customisation for each

F1000 - customisation

eLife - sub articles, wonderful and have DOIs but you never find them in PMC, have to scroll down and down

Mainly coming up as supplementary material - but this is not supplemental material.

Get why everyone does it, but it’s wrong

One file to rule them all or review reports with authors and title, but not searchable field

How can we do this in a way that’s flexible enough to fits everyone’s model but that it’s transparent, archived, found and creditable

Peer reviews - some are in suppl material, some are sub articles. Question - is a peer review or decision letter part of the article or seperate published object?

Is there value in this? No way to track it because so variable and not tagged

Think the value is there, but such a small sample size

Interesting to look at number of downloads or page views of these items?

But have to be identifiable and findable

*Crossref*

Landscape review - link at top

Crossref extended support for peer review history earlier this year

12,000 records from 3 publishers PeerJ and ScienceOpen and Stichting SciPost

Rolled out with consultation with open peer review journals

Generic type of metadata and review specific attributes to provide more granularity and history

Of the existing data they have - know they are getting cited. Of the 12,000 registered

Because they are linked to the article and the often times the preprint is linked to the article, can see the trail in the metadata

Software citation article - 4 preprints then PeerJ then published. Connecting up all these different moments and use cases out of this

The dark character cube paper. Published, and peer review associated with it was cited by the authors of the article in a subsequent publication of theirs. Interesting, and able to see these things

Discoverability is what’s holding back re-use of these reports

ORCID - part of the auto-update service

Will post the review to the associated

Single PDF - could register metadata for it, but trying to make it easy to register the DOI

**Group Synthesis of Individual Accounts**

To think about: if just embarking on publishing peer review reports, what would the minimal information/requirement be?

Other stakeholders we are underrepresented here?

- Commonalities/themes
- Elements of an ideal approach to publishing peer review reports/bundles
- Current pain points
- Red line/absolute requirements

**Group 1**

1. Commonalities/themes (List of objects/technical requirements/author or reviewer permissions/licence of bundle elements)

- Review reports, author response, decision letter
- Different versions of paper as goes through revision
- Capture dates and time of review steps would be useful
- Time to first decision would be good date to record
- Directional linking between paper and process files - should never be able to break that link

1. Elements of an ideal approach to publishing peer review reports/bundles
2. Current pain points

- Systems
- Time - takes too long
- Not automated - the quality of automation might not need to be as high as full article (eg allow some flexibility in layout and display)
- Primary goals - discoverability is important, but being transparent in the first place is the mina
- Structured info about the articles than the rendering of it
- Better to have 10 DOIs with structured metadata directing to one PDF is better than not, but full text would be better

1. Red line/absolute requirements
   - Authorship?Once this is decided, next is easier:
   - Licensing
   - Editors are much more exposed when peer review is open - psychological barrier that is hard to leap
   - But communicate to people nothing bad does happen
   - Reviews could be used for wrong reasons and out of context, so monitoring is necessary to prevent this
2. Ideal - Review process document model
   - Captures timing of process
   - Link to final version +/-intermediate versions
   - Roles and responsibilities clear
   - Licensing info
   - Structure of model is important but also rendering and distribution
   - Process file should be easy to access
   - Need balanced access to reviewers comments and author response - cannot be easily dissociated because taking out of content, but obviously also make separate as well
   - Integration with ORCID
   - Should be citable DOIs - what do we mean by that, for what purpose - ie one reviewer comment that’s negative taken out of context VS 2 great review

Nature Communications - does not publish the decision letters, just the exchange between reviewers and authors to mitigate the editor stress

But, that takes away the point of the editor’s decision making - who makes the decision as to whether it is published.

Devalues the editor?

Nature Communications did what they did to overcome the resistance from the editors

CReDIT

Action? Crossref list of reviewer roles/terms review. Or give to CASRI and ask them to look into it?

Ontology required

Machine readable? XML tagging up of tables etc(complicated documents)

Show value of full text to publishers so they

**Group 2**

- Commonalities/themes
  - Objects - for each version, there may be any number of the following: referee report, editor’s decision letter, author’s rebuttal, manuscript
  - Technical requirements -
  - Reviewer permissions - published reports may be signed depending on the publisher implementation. Permissions need to be established and made clear to reviewer and author in the journal policy.
  - License for the review artifact (separate from the article) - CC-BY
- Elements of an ideal approach to publishing peer review reports/bundles
  - All objects fully tagged to the archive in the same way as the article
  - Fully automated system, seamless exchange from author submission & peer review management system to typesetters/publishing platform to indexers and metadata systems
  - Third party peer review systems are also integrated into this process, whether pre- or post-publication
  - ORCID integration? Downstream tie-ins - Needs a PID?
- Current pain points
  - Existing MTS and publishing platforms - getting data out but also collecting data from submission system
  - Getting the reviews displayed in a user friendly manner on the publishing platform
  - Checks being done - hard to scale
- Red line/absolute requirements
  - Link review artifact to the article
  - Distinguish contributors of the review artifacts where available (i.e., signed) from the author names
  - Easy retrieved
  - Citable (?) Own DOI/PID? Can each component really have their own DOIs, but then you can build all into one PDF

Some publishers cannot give DOIs to the smaller things - eg BMJ and hosting platform cannot do it

**Group 3**

- Commonalities/themes
  - Metadata should include:
    - Journal metadata
    - “Type” - review, decision letter, author reply
    - Decision status
    - link to reviewed content and version of the article
    - Timeline or events
    - Reviewer info - names, identifiers, CoI statements, affiliations, type (start with CrossRef list?)
    - DOI
    - License info
  - Body text of review
  - Ref list
  - Author’s reply - stick in peer reviewer itself? One DOI with multiple authorship?
  - Citable version to tell funder etc, don’t want it cluttered up with other parts of the review process
- Elements of an ideal approach to publishing peer review reports/bundles
  - Pro-open peer review
  - Should be able to do it
  - Just depends on how far publishers want to go
- Current pain points
  - Mandating anything
  - Any optional parts make it more complicated
  - If paper is not open access is there any point of the peer review being open? Does that still add something? Different licenses and issues or quoting from the main paper, what if the review is an annotated PDF of the article
  - External vendors (scholarone)
  - Excluding bad actors - for those publishers who don’t have all open access content, how to encourage them to have some info about the peer review events open or metadata about it
- Red line/absolute requirements

**Top best practice**

Reviews

Responses

Decisions

Metadata for each element - Crossef schema harmonize with JATS?

**Blockers of that best practice**

Publishing the peer review reports

Get rid of the word OPEN - whether it’s free and CCBY of named people exposed, and this is off putting

You can still have blinded peer review and publish the reports.

Transparent is a nicer word, more socially acceptable.

Dissociate from OA

Don’t focus on the access controls to the peer review, but focus on getting them published (whether open or behind access controls)

What would be behind the paywall? Metadata of peer review behind or infront of the paywall

How to cover metadata to expose a review happened but the reviewer wanted to be anonymous and not publish their review.

Eg 3 reviewers, 2 open and one closed - need to make it clear to reader there are 3 reviews that contributed to the decision

Closed review <https://peerj.com/articles/5194/reviews/>

Open Review <https://peerj.com/articles/5039/reviews/>

**Minimal recommendation**

Full text XML desire

Editor roles

DOI

EMBO - getting the metadata out of ejp

Action -minimal example of what to supply to crossref to

- Title nomenclature
- Review date
- Relation (link to article)

**Action**: Jennifer Lin to provide a reference XML document of the most complicated and full

Required will be highlighted

**Action:** all review this output

Biggest hurdle with platform providers - getting any metadata

Ask for all at first bat

Crossref will carry on with the schema until it changes

**Vendor hosts:**

HighWire

Atypon

Continuum

SilverChair

PeerJ

F1000

**Submission systems:**

EJP

ScholarOne

RiverValley

Aries

COKO

BenchPress

MECA - portable peer review

Attribution system to the journal who ran the review?

**Wrap up**

What will we do and what are the top priorities

Write this up as a paper

Refine some of the concepts into recommendations or learnings

Take things back to do individually

Engage others

Schematron to define subset

For the things sketched out by Jeff’s group - write a schematron to validate what this team would approve

Make some dummy JATS XML

Description of what needs to be a peer review

Use case in the paper - lowest common denominator

But paper needs to reach the editorial crowd

Supplemental material - get away from this

Title

Year

Relationship

**Actions:**

Melissa/Jo Tidy up the document and give it some structure - By end of August

Set up some sessions for co-writing in Sept

Publish in Oct

Jennifer Lin XML document - By end of next week

Jeff - JATS models - separate documents;sub articles; supplemental files - By end of August

Phil - Use cases/stories - complexities

Thomas - use cases

All - compelling peer review stories

Prototype how this could work? Event like talking to Force11?
