# Supplementary material for: Publishing peer review materials
Source: F1000Res. 2018 Oct 17;7:1655. [Version 1] doi: 10.12688/f1000research.16460.1 (PMC6206614; doi:10.12688/f1000research.16460.1)
Supplement: Peer review history

# Peer review: Referee response for version 1

**DOI:** 10.5256/f1000research.17992.r39584

**Published:** 2018-10-29

**Authors:** Tony Ross-Hellauer

**Affiliations:** 1 Graz University of Technology, Graz, Austria; 2 Know-Center, Graz, Austria

**“Publishing peer review materials” **reports the outcomes of a 2018 workshop convened with stakeholders engaged in the publication and dissemination of peer review report, to work towards consensus on “how to represent these materials in the JATS and Crossref data models to facilitate the coordination and discoverability of peer review materials”.

 The article is valuable for understanding the range of publisher workflows in operation for publishing peer review at present. As such, it both makes the case for the need for standardisation of description and proposes a draft JATS XML proposal designed to aid depositing to Crossref. The proposals are pragmatic and reasonable, reflecting the variety of workflows in operation at present and the possibilities for timely transition towards a more standardised system.

 This first version clearly indicates that it is a “draft” to “seek feedback on these initial recommendations” – but despite its provisional nature, I believe it is ready to be indexed already in this version, as it is a sufficiently complete record of the discussion to this point.

 The article makes very clear how much work lies ahead, and that this document is just a starting point. Given its purpose, I don’t believe formal peer review is actually necessary here – as a working proposal released to the community for feedback, the views of each member of the community are equally important. I hope my comments will be received as just one more voice in that conversation, and given equal weight with views shared by other channels.

 I agree with Reviewer 1’s comments regarding useful ways to enhance the level of detail here. To this I would only add that it would be useful to describe why the JATS elements under the sub-heading “Not allowed” are so - why must they not appear in review documents?

 Other than this, I would only note some further thoughts for how these discussions could develop in the future. None of these issues need necessarily be considered within this present article, but I record them here in hopes of continuing the conversation:

  

- **Links to review policies: **To understand the context of the review it would be helpful to have some lasting link to review policies under which the review was conducted. For example, what criteria were the reviewer asked to consider? Was it single/double-blind or open identities review? If the latter, were reviewer identities revealed to the author (and if so at what stage)? This information could be made available via descriptive metadata fields in this schema, or via persistent links to policy descriptions.
- **From “is” to “ought”: **I understand it is not the scope of this document to prescribe practices, but to ensure pragmatic routes to interoperability of metadata description. Hence, I understand that the “topic of licensing of the peer review materials was discussed at the workshop but ultimately left out of the remit of this group because the JATS tagging schema would allow for different licensing information to be added for these items or to retain that of the main article, as a publisher chooses”. Nonetheless, I think as a community some best-practice guidelines would be helpful to ensure the interoperability and re-usability of this content. This point goes more generally for other best-practice issues (e.g., assignment of DOIs). In the “Future/next steps” section you note that discussions should continue via other fora – here, I wonder if there is a need for a formal working group hosted by, for example, Force11, to work towards consensus on such topics. (This could also potentially link up with the PEERE group’s work on peer review data-sharing).
- **Reviewer roles: **We may in future see more stratification of reviewer roles and it would be interesting to have recorded exactly what parts of a manuscript each reviewer had considered (methodology, stats, analysis, etc.). Also, if any computational tools had been applied to check, e.g., statistics. Here it would be useful to have a standard typology of such roles.
- **Other review materials: **There are a multitude of venues for “post-publication” review – including journal article comment sections, third-party platforms like publons or pub-peer, or even individual blogs, etc. It would be useful to have formal, persistent links between such materials and the article in question so that readers may more easily place the article in the context of a continuing discussion. I imagine Crossref event data will be useful for this, but I wonder if there would be value in third-party platforms also applying this schema (suitably extended/adapted) to represent these links in a more formal way?

I have read this submission. I believe that I have an appropriate level of expertise to confirm that it is of an acceptable scientific standard.

# Peer review: Referee response for version 1

**DOI:** 10.5256/f1000research.17992.r39586

**Published:** 2018-10-22

**Authors:** Jessica K. Polka

**Affiliations:** 1 ASAPbio, San Francisco, CA, USA

In “Publishing peer review materials,” Beck and coauthors present the outcome of a workshop that assessed the current state and best practices for the technical representation of peer review material and metadata. The report is well-organized and proposes guidance on XML tagging that the group hopes will be adopted by JATS4R. This article is an important contribution to the conversation about increasing transparency in peer review and will facilitate its development as a valued, linked, and preserved class of scholarship.

 I don’t feel that any of the following comments need to be addressed before the manuscript is indexed in PubMed, but I do believe that the report could be strengthened by attention to these areas:

**Presentation**

- This piece would benefit from more illustration of workshop outputs. For example, is there a file you can point (or embed inline) that demonstrates best practices for tagging?
- In a similar vein, there is great information in Supplementary File 2. Can the process information be used to generate an in-text table?

**XML proposal**

*Note that I lack practical experience with JATS.*

- Could the tag also be used to identify student or postdoc co-reviewers?
- Is intended to represent the publication date of the review (presumably the same as the article) or the (in this case probably more useful to readers) date that the review was submitted? Or is that captured in ? Clarification would be helpful.
- Why is disallowed? I would like to imagine a future in which peer review is recognized as a valuable scholarly activity, something that funders would like to know they are supporting.

**Text edits**

- Please define “BMA.”
- “Past experience has taught us that making content available is just the first step toward increasing transparency.” What does this refer to?
- “The term manuscript does not map to anything we’ve discussed.” What does this mean?

I have read this submission. I believe that I have an appropriate level of expertise to confirm that it is of an acceptable scientific standard.
